# Book Notices

**Published:** 1878-11-20

**Authors:** 


### BOOK NOTICE.

Transactions of the Missippi State Medical Association—vol. IX. Being the eleventh annual session held at Jackson, Mississippi, April 1878, with the roll of members, and reports of medical topics.

This is a very neat and creditable work, of 167 pages. The address of B. A. Vaughn, M. D., President, devoted to Sanitary Science, etc., is able, instructive and interesting.

The Annual Oration by J. E. Halbert, M. D., presents an interesting resume of the history of medicine.

Then comes in succession, the following papers, some of which are decidedly able, and all of which will well repay perusal ; to wit:

Salicylic Acid, by Dr Wirt Johnston ; Treatment of Diphtheria, by Dr P F Whitehead ; A Case of Criminal Poisoning with Arsenious Acid, by Dr S V D Hill ; Case of Suppuration of Antrum Highmorianum, by Dr J R Barnett ; Syphilis in the Negro, as differing from the syphilis in the White Race, by Dr Wm Powell, Grenada ; Hydrophobia, by Dr H J Ray ; Posture in Treatment of Colic, by Dr D L Phares ; Croupous Pneumonia, by Dr J W Holman ; Malaria. What is it, how produced, and how prevented, by Dr Thos. Bryan ; Chronic Catarrh, by Dr R G Wharton ; Case of Poisoning by Hydrate Chloral and Camphor, by Dr H Hanslow ; Chloral Hydrate in Obstetrics, by Dr J E Halbert ; Wound of Plantar Arch, Treated by Compression, by Dr H Hanslow ; Treatment of Cholera, by Dr B F Kittrell ; Epidemic Cerebro-Spinal Meningitis, by Dr E W Hughes ; Early Management of the Infant, by Dr J T Parker ; Typhlitis, by Dr A H Cage ; Surgical History of Mississippi, by Dr W W Hall ; Report of Committee on Necrology.

---

Transactions of the Medical and Chirurgic-Faculty of the State of Maryland at its 18th Annual Session Held at Baltimore, Apr, 1878.

A well gotten up volume of over 200 pages, containing the opening address of Dr. A. B. Arnold, on Homœopathy. A report of fifty-tw- successful cases of lithotomy, by Allen P, Smith, M. D. Apyretics and Antipyretics in Fever, by John S. Lynch, M. D.

Chloroform in Obstitrics, by P. C. Williams, M. D.

Report On Materia, Medica and Chemistry, by J. E. Atkinson, M. D.

Spontaneous Generation, by Prof. F. Donaldson M. D.

General Paralysis of the Insane by J. D. Thompson, M. D.

Removal of Naso-Pharyngeal Polypus by Temporary Depression of Both Upper Jaws, by S. McLane Tiffany, M. D.

The New Treatment of Chorea, by John Van Bibber, M. D.

Report of a Case of Exophthalmus, by Joseph A. White, M. D.

A Case of Double Vagina, by A. F. Erich, M. D.

The above papers are in the main interesting and valuable; but we have not space for special mention of their merits.

Pocket Therapeutic and Dose Book with clasification and explanation of the action of medicins etc., by Morse Stewart Jr. B. A., M. D. Detroit, Mich. Price 50ct.

---

Notes Taken from a Lecture by Dr Manuel Dagnimo, at the Medical University of Caracas, Capital of Venezula on the Treatment of Yellow Fever. Translated into English by Dr, Antonio De Tejada, of New York.

A Series of American Clinical Lectures, edited by R. C. Seguin, m. d.

Operation for Closeure of Cleft of Hard and soft Palate. By A. Vanderveer, M. D., Professor of Principles and practice of Surgery, etc., New York. G. P. Putnam & Sons.

Diet and Hygiene in Diseases of the Skin, by L. Duncan Bulkley A. M., M, D. Fellow of New York Academy of Medicine, Physician to the Skin Department, Demilt Dispensary New York etc, etc

On the Use of the Solid Rubber Bandage, in the Treatment of Eczema and the Ulcers of the Leg, by L. Duncan Bulkley, A. M. M. D., etc.

These papers by Dr. Bulkley are highly interesting and instructive.

---

### THE MALT PREPARATIONS.

Te Malt preparations are coming into general use, and have proven exceedingly valuable to the profession. Samples from Tromer's Extract of Malt Co., have been tested in our practice with gratifying results. The article manufactured by this enterprising and reliable company has been shown by analysis as well as by practical results to be fully equal to the best German make, while the variety and excellence of the combinations which they present are exceedingly useful and convenient to the practitioner. Three different combinations may be seen in their advertisements in this journal. We invite attention also to an article on this subject in our present number, read before a Medical Society.

---

MCKESSON & ROBBINS, of New York, wholesale importers and exporters of drugs, anounce that they received a medal for their exhibit at Paris.

They did not exhibit their Gelatine-Coated Pills for the reason that, they had already received highest medals both at Vienna and Philadelphia, and, as they desired to be known in their relations abroad, as exporters, as well as importers and manufacturing chemists, they confined their exhibits entirely to crude indigenous drugs and essential oils.

---

### MESSRS. REED & CARNRICK.

We invite attention to the new and interesting advertisement of Messrs. Reed & Carnrick which commences with the present number. As Manufacturing Pharmacists they rank among the very best houses in America. We have been kindly promised a sample ef their preparation of Maltine which we hope to test and report upon hereafter.

